# Immune Checkpoint Signatures Reveal Stage-Specific Biomarkers for High-Activity Multiple Sclerosis

**DOI:** 10.3390/ijms27041907

**Published:** 2026-02-16

**Authors:** MariPaz López-Molina, Gabriel Torres Iglesias, Laura Vidal, Nerea Díaz Gamero, Álvaro Sánchez-Pascual, Beatriz Chamorro, Roberto Lozano-Rodríguez, Gonzalo Sáenz de Santa María-Diez, Julia del Prado-Montero, Eduardo López-Collazo, Exuperio Díez-Tejedor, Fernando Laso-García, María Gutiérrez-Fernández, Laura Otero-Ortega

**Affiliations:** 1 Neurological Sciences and Cerebrovascular Research Laboratory, Department of Neurology, Neurology and Cerebrovascular Disease Group, Neuroscience Area of Hospital La Paz Institute for Health Research IdiPAZ, La Paz University Hospital, Universidad Autónoma de Madrid, 28046 Madrid, Spain; mplm1995@gmail.com (M.L.-M.);; 2 Immune Innate Response Group, La Paz Hospital Institute for Health Research IdiPAZ, La Paz University Hospital, 28046 Madrid, Spain; 3 Tumor Immunology Laboratory, La Paz Hospital Institute for Health Research IdiPAZ, La Paz University Hospital, 28046 Madrid, Spain; 4 Centro de Investigación Biomédica en Red (CIBER) of Respiratory Diseases (CIBERES), 28029 Madrid, Spain; 5 Translational Stroke Laboratory (TREAT), Clinical Neurosciences Research Laboratory (LINC), Health Research Institute of Santiago de Compostela (IDIS), 15706 Santiago de Compostela, Spain

**Keywords:** highly active multiple sclerosis, immune checkpoints, multiple sclerosis, treatment choice, immune landscape

## Abstract

The early identification of patients with highly active multiple sclerosis (HAMS) is crucial for guiding therapeutic decisions and initiating high-efficacy treatment strategies. This study aimed to characterize peripheral immune profiles that can distinguish between patients who are candidates for intensive therapy at disease onset and in later stages. Using spectral flow cytometry, we identified distinct immune signatures to differentiate early-stage patients from those with refractory, long-standing disease. In newly diagnosed individuals, decreased herpesvirus entry mediator (HVEM) expression on effector T helper (Th) cells distinguished HAMS from non HAMS cases. In contrast, patients with therapeutic resistance exhibited reduced CD28 expression on naïve regulatory and CD8^+^ T cells. Disability progression was associated with elevated HVEM on classical monocytes, decreased CD70 on CD56^bright^ natural killer cells (NK), and lower programmed cell death protein 1 (PD-1) expression on memory Th cells. Notably, CD28 expression on terminal effector CD8^+^ T cells and HVEM levels on plasmablasts emerged as strong predictors of progression independent of relapse activity, while higher PD-1 memory Th cell frequencies predicted clinical stability. This study identifies two panels of immune biomarkers: one distinguishing candidates for early high-efficacy intervention, and another defining patients with refractory disease. The immunological landscape of HAMS evolves across disease stages. In addition, we defined progression-associated markers detectable at the outset of follow-up, enabling the timely recognition of patients at heightened risk of disability accumulation, discriminating between neurodegeneration-driven and inflammation-driven mechanisms of progression.

## 1. Introduction

Multiple sclerosis (MS) is a neurological disease driven by immune system dysfunction that targets the central nervous system (CNS), impacting approximately 2.8 million individuals globally [1]. MS arises from a combination of genetic susceptibility and environmental triggers. This interaction disrupts immune tolerance, particularly toward myelin components in the CNS. As a result, autoreactive T cells, along with B cells and monocytes, breach the blood–brain barrier and infiltrate neural tissues in the brain and spinal cord. Within the CNS, these immune cells produce cytokines and autoantibodies that activate resident glial cells, astrocytes and microglia, which trigger programmed cell death in oligodendrocytes. The ensuing immune-mediated inflammation causes demyelination, leading to progressive axonal damage and neuronal loss [2].

The clinical course of MS is classified into two different phenotypes, relapsing-remitting MS (RRMS) and progressive MS (PMS). RRMS is the most common phenotype, characterized by acute episodes of neurological deficit followed by periods of remission. Approximately 50% of these patients develop progressive disability, classified as secondary progressive MS (SPMS). Only 15% of patients develop a progressive phenotype from the onset, classified as primary progressive MS (PPMS) [3]. There is a subgroup of RRMS patients who have a highly active disease course (HAMS) marked by frequent extensive inflammatory activity evident through severe relapses, magnetic resonance imaging (MRI) scans, and a rapid accumulation of disability [4]. These patients often experience significant clinical deterioration despite treatment with standard disease-modifying therapies (DMTs), necessitating more effective therapeutic strategies to control disease activity and prevent further neurological damage [5,6,7].

The lack of consensus regarding a standardized definition for HAMS complicates diagnosis. This adds to the fact that the variability in disease presentation and progression makes it difficult to distinguish HAMS from other forms of MS based on clinical symptoms alone [8]. Therefore, there is a critical need for reliable biomarkers that accurately identify patients with HAMS and would enable the early initiation of high-efficacy treatments, tailored to halt the rapid progression and high activity of the disease, improving patient outcomes.

Monitoring immune system activity in patients with MS may offer valuable insights into the aberrant inflammatory responses driving the development of HAMS. By assessing immune cell subsets and their immune checkpoint (IC) expression, clinicians may detect subclinical immune dysregulation not captured by conventional clinical or radiological assessments, which would help design an immune panel to distinguish patients who have HAMS. This study aims to characterize peripheral immune system patterns to identify patients at risk for HAMS and guide early therapeutic intervention. We also aim to analyze whether immune biomarkers remain stable throughout the stages of HAMS or if distinct immunological signatures emerge over time, from disease onset to long disease with multiple therapeutic failures. This strategy may serve for stratifying patients and optimizing the timing and efficacy of therapeutic strategies.

## 2. Results

**Figure 1:**
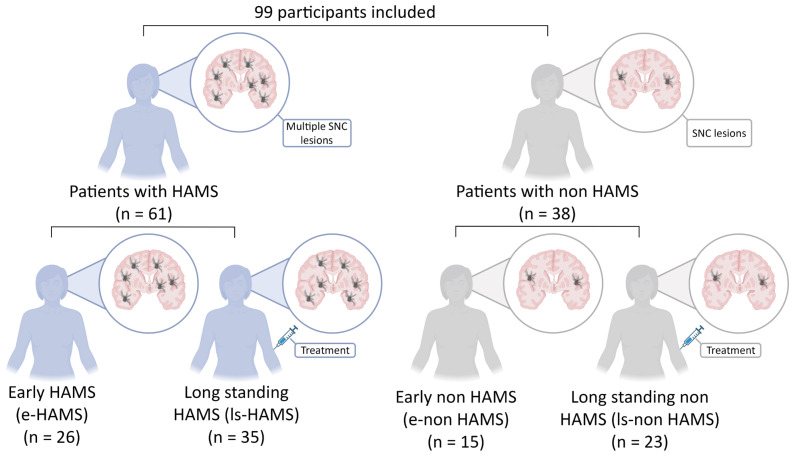
Flow chart of study design. Schematic representation of the study design including 99 patients with relapsing-remitting multiple sclerosis (RRMS). Among them, 61 were classified as having high disease activity (HAMS) and 38 as non HAMS. The HAMS group included 26 newly diagnosed, treatment-naïve, early-stage patients (e-HAMS) and 35 patients with long-standing disease and previous treatment failures (ls-HAMS). The non HAMS group included 15 newly diagnosed, treatment-naïve, early-stage patients (e-non HAMS) and 23 with long-standing disease MS patients (ls-non HAMS). Created in BioRender. Otero, L. (2026), https://app.biorender.com/illustrations/698b0bba3f7e03c2e0ea506c.

The study enrolled 99 patients with RRMS including 61 patients with HAMS and 38 with non HAMS (Figure 1). Patient characteristics are summarized in Table 1. Neurofilament (NfL) levels further supported disease stratification, with higher levels detected in HAMS patients versus non HAMS patients (11.33 ± 6.51 ng/mL vs. 7.67 ± 4.57 ng/mL; *p* = 0.010).

### 2.1. Unsupervised t-SNE Analysis Reveals Immune Cell Heterogeneity in Early-Stage MS Patients and Those with Long-Standing Disease

**Figure 2:**
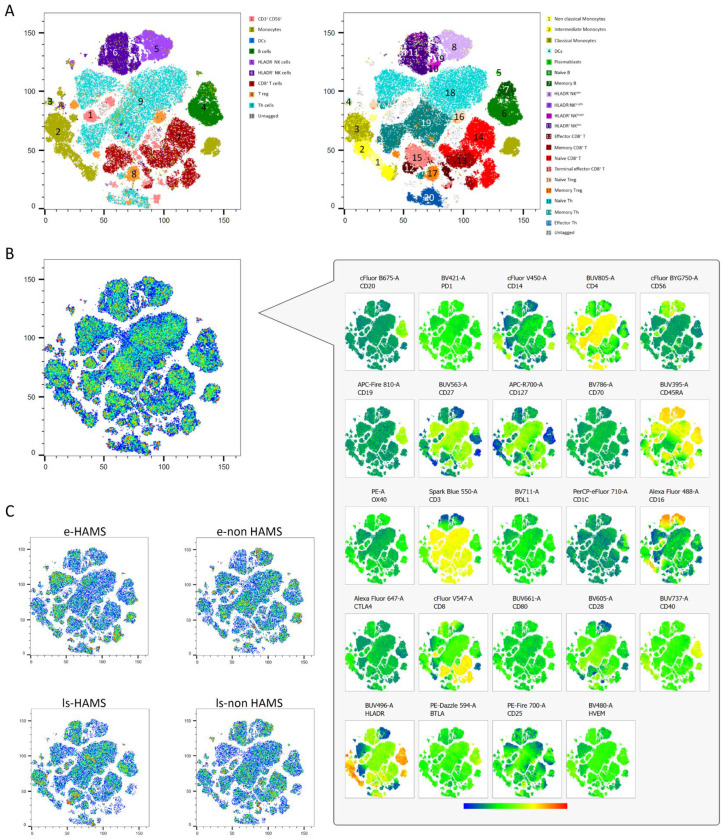
High-dimensional immune profiling. (**A**) t-SNE projection of PBMCs from 40 donors: 10 early high-activity MS (e-HAMS), 10 early non-high-activity MS (e-non HAMS), 10 long-standing high-activity MS (ls-HAMS), and 10 long-standing non-high-activity MS (ls-non HAMS), each downsampled to 10,000 events. FlowSOM clustering was applied and overlaid on the t-SNE map to demonstrate consistency between supervised and unsupervised analytical approaches, as well as to visualize the overall cohort distribution. The left panel displays nine main immune cell lineages, while the right panel shows 20 finer immune subpopulations derived from lineage-specific reclustering. Clusters that do not fit standard immune definitions are shown in gray (ungated). (**B**) Overlay plots and corresponding heatmaps illustrate marker intensity (fluorochrome expression) across the 20 identified immune populations, using concatenated events from all donors. (**C**) Density plots highlight differences in immune cell population distribution among the four study groups, revealing immunophenotypic divergence associated with disease activity and disease duration. Untagged: computationally classified cells that do not appear as a visible cluster in the t-SNE plot.

Unsupervised T-distributed stochastic neighbor embedding (t-SNE) analysis was performed on peripheral blood samples from 40 RRMS patients, comprising 10 early high-activity MS (e-HAMS), 10 early non-high-activity MS (e-non HAMS), 10 long-standing high-activity MS (ls-HAMS), and 10 long-standing non-high-activity MS (ls-non HAMS), providing a representative distribution of the four clinical subgroups. The ten patients in each group were randomly selected to avoid bias. Seven major immune lineages, monocytes, B cells, NK, dendritic cells (DCs), CD8^+^ T cells, Treg, and Th cells, were identified and independently reclustered, yielding a total of 21 distinct immune cell populations (Figure 2A). Two specific clusters (11 and 10) remained ungated in conventional lineage definitions and were further characterized via comprehensive marker screening (Figure 2B). These clusters displayed phenotypic features of NK cells (CD16^+^CD56^+^) but also expressed CD45RA^+^ and Human Leukocyte Antigen (HLA)DR^+^, suggesting a naïve yet activated immune phenotype renamed as HLADR^+^ NK^dim^ and HLADR^+^ NK^brigth^. The spatial proximity to NK cells on the t-SNE plot supported its inclusion within the NK lineage.

This unsupervised approach allowed the assessment of immune cell abundances across disease stages. Notable differences in the density of major immune subsets were observed between the HAMS and non HAMS groups. Overall, a higher percentage of effector CD8^+^ T cells and naïve B cells were observed in long-standing patients compared to early-stage individuals. Within the early-stage cohort, e-non HAMS patients exhibited the lowest abundance of monocytes, together with a reduction in terminal effector CD8^+^ T cells. Regarding long-standing patients, those without high disease activity exhibited a higher abundance of Treg cells and a lower proportion of naïve Th cells compared to ls-HAMS (Figure 2C). This unsupervised analysis served exclusively as an exploratory and qualitative visualization approach rather than for statistical inference; however, its results were similar to the findings of the supervised analyses.

**Figure 3:**
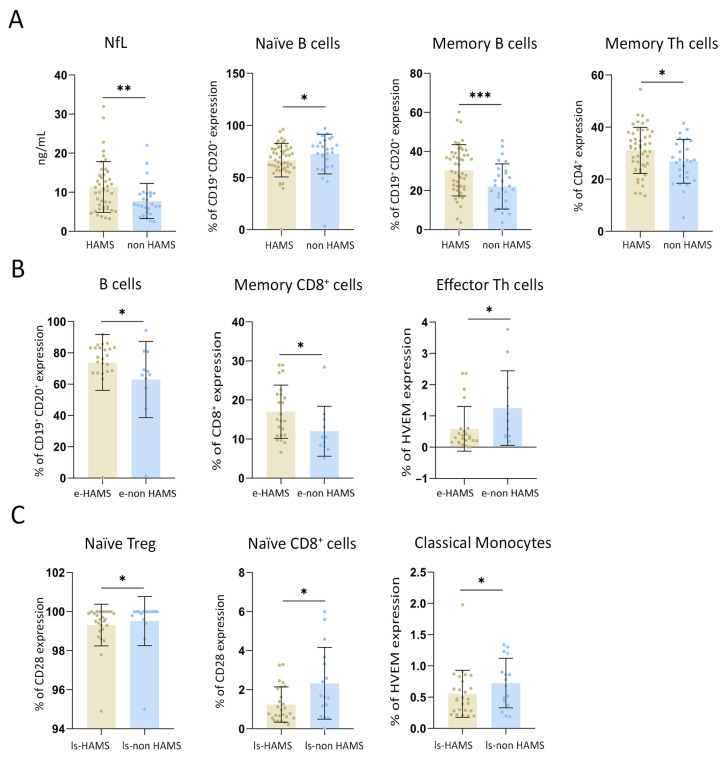
Differential immune profiles and immune checkpoint expression across MS subgroups. (**A**) Comparison of immune cell populations and plasma neurofilament light chain (NfL) levels between HAMS and non HAMS groups across the entire cohort. (**B**) Frequencies of immune cell subsets and expression levels of immune ICs in newly diagnosed, treatment-naïve, early-stage patients, comparing those classified as HAMS versus non HAMS. (**C**) Differences in IC expression levels in long-standing disease and previous treatment failures patients, comparing those with HAMS to those without. Abbreviations: early high-activity MS (e-HAMS), early non-high-activity MS (e-non HAMS), long-standing high-activity MS (ls-HAMS), and long-standing non-high-activity MS (ls-non HAMS). Statistical significance was defined as follows: *, *p* < 0.05; **, *p* < 0.01; ***, *p* < 0.001.

By a supervised approach, significantly lower levels of naïve B cells were found in HAMS patients compared to non HAMS patients (66.73 ± 16.13% vs. 72.50 ± 19.11%; *p* = 0.044). Conversely, high levels of memory B cells were detected in HAMS patients (30.39 ± 13.14% vs. 22.12 ± 11.76%; *p* = 0.001). Moreover, memory Th cells were shown to be significantly higher in HAMS compared to non HAMS patients (31.08 ± 8.84% vs. 26.86 ± 8.40%; *p* = 0.044) (Figure 3A).

### 2.2. Immune Signatures Distinguish HAMS at Diagnosis in Early-Stage MS Patients

To evaluate whether immunophenotypic markers can identify HAMS patients at disease onset, we analyzed their immune profiles (Figure 3B). Patients classified as e-HAMS exhibited significantly higher proportions of B cells (75.85 ± 17.82% vs. 62.94 ± 24.34%; *p* = 0.043) and memory CD8^+^ T cells (17.00 ± 6.38% vs. 12.01 ± 6.39%; *p* = 0.028) compared to e-non HAMS patients. Furthermore, expression of HVEM on effector Th cells was markedly increased in e-non HAMS patients relative to e-HAMS (1.25 ± 1.19% vs. 0.59 ± 0.71%; *p* = 0.031), suggesting an early immunological signature associated with high activity. These findings point to reduced HVEM expression as early biomarkers of true high-risk disease, supporting their use in guiding the prompt initiation of high-efficacy therapies at the time of diagnosis.

### 2.3. The Immunophenotypic Profile Identifies Therapeutic Resistance in Long-Standing MS and Recognizes Candidates for High-Efficacy Interventions

We first ensured that the different DMTs had undergone a sufficient washout period, specified in the product information, to allow the restoration of the patient’s immune system at the time of blood analysis, and that none of the treatments had left an immunological imprint. We then investigated whether there were differences in immune cell populations and IC expression among patients with different prior treatments. Importantly, this analysis was performed only for those treatment groups with enough patients to allow a meaningful comparison. We found no significant differences in these variables (Table 2).

To investigate the immune features associated with therapeutic resistance in long-standing MS, we compared IC expression between long-standing MS patients with low (ls-non HAMS) and high (ls-HAMS) disease activity. Notably, there was a significantly higher expression of the co-stimulatory molecule CD28 in both naïve Treg and naïve CD8^+^ T cells in the ls-non HAMS group compared to the ls-HAMS group (99.52 ± 1.26% vs. 99.31 ± 1.07%, *p* = 0.040, and 2.32 ± 1.83% vs. 1.24 ± 0.90%, *p* = 0.049, respectively). This suggests the potential exhaustion of early T cell activation in patients with refractory disease. Additionally, HVEM showed reduced expression on classical monocytes from ls-HAMS patients compared to ls-non HAMS patients (0.55 ± 0.38% vs. 0.73 ± 0.40%; *p* = 0.037) (Figure 3C). These findings reveal an immunophenotypic signature in patients with long-standing, treatment-refractory MS, supporting their identification as candidates for high-efficacy therapeutic strategies.

### 2.4. Immunophenotypic Markers Identify True Poor-Prognosis Patients

**Figure 4:**
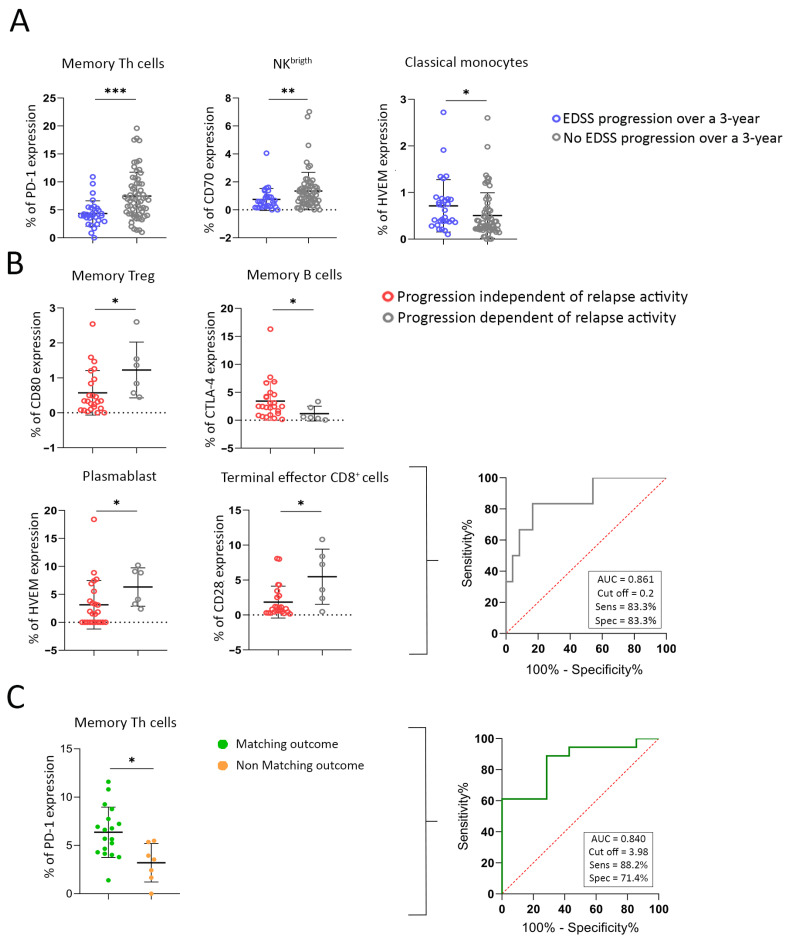
Expression of immune checkpoints predicts disability progression and refines prognostic classification in MS. (**A**) Comparison of IC expression levels between patients with and without EDSS progression at 3 years. (**B**) IC expression differences between patients who experienced progression independent of relapse activity (PIRA) and those who did not. A logistic regression model was used to predict PIRA, and internal validation was performed using receiver operating characteristic (ROC) analysis. HVEM expression on plasmablasts and CD28 expression on terminal effector CD8^+^ T cells (gray curves) showed predictive value. (**C**) To enhance prognostic precision beyond initial clinical classification, we assessed whether IC expression could identify patients originally categorized as non HAMS who nonetheless experienced disability progression over 3 years, patients typically considered candidates for lower efficacy therapies. Based on PD-1 expression on memory Th cells being significantly lower in those who progressed compared to those who remained stable, ROC analysis revealed that PD-1^+^ memory Th cell frequency had strong predictive power for disease stability (AUC = 0.840). A threshold of 3.98% provided 83.2% sensitivity and 71.4% specificity (green curve), supporting PD-1 as a promising biomarker for identifying truly stable patients. EDSS = Expanded Disability Status Scale. Non Matching outcome = Patients initially classified as non HAMS who experienced disability progression over a 3-year period. Matching outcome = Patients initially classified as non HAMS who nevertheless experienced disability progression over a 3-year period. Statistical significance was defined as follows: *, *p* < 0.05; **, *p* < 0.01; ***, *p* < 0.001.

Current clinical practice defines patients with HAMS based on early clinical indicators of poor prognosis. However, these criteria may not always accurately predict long-term outcomes. To address this limitation, we examined whether blood-based immunophenotypic markers are more precise in identifying patients who will experience disability progression over time. We found that specific IC expression patterns were significantly associated with expanded disability status scale (EDSS) progression at 3 years. HVEM expression on classical monocytes was significantly higher in patients who progressed compared to those who did not (0.71 ± 0.56% vs. 0.51 ± 0.49%; *p* = 0.044), indicating early innate immune activation as a predictor of disability accumulation. Conversely, CD70 expression on NK^brigth^ cells was elevated in patients without progression (1.34 ± 1.34% vs. 0.74 ± 0.78%; *p* = 0.026). Additionally, in the adaptive compartment, patients without progression had a significantly higher PD-1 expression on memory Th cells than those who progressed (7.47 ± 4.26% vs. 4.33 ± 2.29%; *p* = 0.001) (Figure 4A). These findings indicate that blood immunophenotyping can uncover biological signatures associated with a true poor prognosis in MS, thus truly requiring early escalation to high-efficacy therapies.

### 2.5. CD28 Expression on Terminal Effector CD8^+^ T Cells and HVEM in Plasmablasts as Predictors for Progression Independent of Relapse Activity

We next aimed to determine whether, among patients exhibiting disease progression, the expression profiles of ICs differ between those classified as having progression independent of relapse activity (PIRA) and those with relapse-associated worsening (RAW), with the objective of discriminating between neurodegeneration-driven and inflammation-driven mechanisms of progression.

Patients exhibiting PIRA showed decreased CD80 levels in memory Tregs compared to those with RAW (0.57 ± 0.63% vs. 1.23 ± 0.80%, *p* = 0.026). Similarly, lower expression levels were observed in patients with PIRA for HVEM in plasmablasts (3.13 ± 4.33% vs. 6.32 ± 3.43%, *p* = 0.020) and CD28 in terminal effector CD8^+^ T cells (1.84 ± 2.28% vs. 5.48 ± 3.95%, *p* = 0.027). Conversely, cytotoxic T-lymphocyte-associated protein 4 (CTLA-4) expression on memory B cells was significantly higher in patients with PIRA compared to those with RAW (3.43 ± 3.45% vs. 1.19 ± 0.54%, *p* = 0.036) (Figure 4B).

Given the large number of ICs identified to predict PIRA, a multivariate model was developed to assess which markers are truly important. Using backward stepwise Wald regression, CD28 expression on terminal effector CD8^+^ T cells and HVEM expression on plasmablasts were identified as robust predictors of PIRA with an OR (95% CI) of 1.565 (1.081, 2.265), *p* value 0.018, and 1.244 (0.977, 1.582), *p* value 0.076, respectively (Table 3). The model was internally validated using a receiver operating characteristic curve (ROC), which showed good model fit (area under the curve (AUC) = 0.86, cut off = 0.2, sensitivity = 83.3% and specificity = 83.3%) (Figure 4B).

**Formula**

$$\log\left(\frac{p}{1-p}\right)=-4.005+0.448\times CD28\;\left(on\;terminal\;effector\;CD8^{+}cells\right)\;+0.218\times HVEM\;(on\;plasmablast)$$

### 2.6. PD-1 Expression on Memory Th Cells as a Biomarker of True Prognosis of Disease Stability

Among the patients whose disability progressed over 3 years, only 39.5% were initially diagnosed with HAMS, based on clinical and radiological markers of poor prognosis. Consequently, only these patients received high-efficacy treatment. In contrast, a proportion of the patients who experienced progression had been misclassified at baseline as having non HAMS, using the same criteria. To refine prognostic accuracy beyond baseline clinical classification, we examined whether IC expression could differentiate patients initially classified as non HAMS who nevertheless experienced disability progression over a 3-year period as candidates for low-efficacy treatments. Based on prior observations showing that PD-1 expression on memory Th cells was significantly lower in patients who progressed over 3 years compared to those who remained clinically stable (3.20 ± 1.99% vs. 6.15 ± 2.61%; *p* = 0.038), an ROC analysis was performed. The percentage of PD-1^+^ on memory Th cells demonstrated a strong predictive value for stability, with an AUC of 0.840. A threshold of 3.98% yielded a sensitivity of 83.2% and a specificity of 71.4% for predicting non-progression (Figure 4C). These results highlight PD-1 expression on memory Th cells as a promising biomarker of true clinical stability, capable of distinguishing patients with favorable long-term outcomes, even when initial clinical assessments suggest otherwise.

## 3. Discussion

Identifying patients with HAMS at an early stage remains a clinical priority, as these individuals benefit from the early initiation of high-efficacy therapies to reduce inflammatory activity and delay irreversible disability progression. In this study, our aim was to identify reliable immune biomarkers capable of stratifying high disease activity in both newly diagnosed patients and those with long-standing disease and multiple treatment failures.

Among newly diagnosed early-stage patients, we observed an immunological signature characterized by increased frequencies of memory CD8^+^ T cells and B cells. This phenotype likely reflects a heightened state of immune activation and antigenic experience early in the disease course. Memory CD8^+^ T cells, known for their cytotoxic potential and rapid recall responses [9], suggest persistent or recurrent CNS antigen exposure, entailing their expansion. In parallel, the elevated proportion of B cells, key players in antigen presentation and antibody production, may further amplify the immune response and contribute to CNS damage through mechanisms such as epitope spreading and antibody-mediated injury [10]. Furthermore, we identified a reduced expression of HVEM on effector Th cells in HAMS patients. HVEM is involved in the regulation of co-stimulatory and co-inhibitory signaling; its downregulation may impair checkpoint pathways that normally temper T cell activation, thereby favoring unchecked pro-inflammatory responses [11]. In addition, HVEM expression on effector Th cells correlated with its binding to BTLA, its inhibitory ligand, further supporting its role in the regulation of T cell inhibition [9,12]. This immune dysregulation aligns with the elevated sNfL levels observed in these patients, reflecting the ongoing axonal injury and reinforcing the concept of early aggressive immunopathology [13].

Interestingly, when we analyzed patients with long-standing disease who had failed multiple treatment lines, we identified a markedly different immune profile. These individuals showed decreased expression of CD28 on naïve CD8^+^ T cells and naïve Tregs, as well as reduced HVEM expression on classical monocytes. From an immunological standpoint, the loss of CD28, a key co-stimulatory molecule required for full T cell activation, may reflect chronic antigenic stimulation leading to T cell exhaustion or senescence [14]. This phenomenon has been associated with persistent inflammation and reduced responsiveness to both antigens and therapy. Similarly, the downregulation of HVEM on monocytes may impair the delicate balance of activation and inhibition within innate immune pathways, potentially contributing to sustained low-grade inflammation and ineffective immune regulation [15]. Notably, HVEM expression on monocytes has been linked to inflammatory activation [16], whereas its reduction in ls-HAMS could indicate monocyte exhaustion.

These findings support the critical concept that biomarkers of disease activity are dynamic and stage-dependent. The immune mechanisms driving early, aggressive disease may differ substantially from those operating in treatment-refractory cases with longer disease durations. This distinction is essential when designing and applying treatment biomarker-based strategies in clinical practice. A biomarker predictive of early HAMS may lose sensitivity or relevance in the context of chronic, therapy-resistant disease, where immune exhaustion becomes predominant.

It is noteworthy that, in our study, a substantial proportion of patients who experienced disability progression during follow-up had not been identified as HAMS at baseline following clinical criteria. This misclassification likely contributed to the delayed initiation of high-efficacy therapies in these individuals, potentially impacting long-term outcomes. To reduce this diagnostic error, we aimed to identify an immunological biomarker panel predictive of long-term disability progression. Specifically, we analyzed immune profiles in patients with confirmed progression, as indicated by sustained EDSS increases during a three-year follow-up. Identifying such cases early through immunological signatures could significantly enhance diagnostic accuracy. Our analysis revealed that patients who experienced disease progression exhibited a significantly reduced expression of PD-1 (a key inhibitory receptor involved in T cell exhaustion and peripheral tolerance) on memory Th cells that may reflect a failure to appropriately limit T cell activation [17], potentially fostering a sustained inflammatory response within the CNS. Similarly, decreased CD70 expression on NK^brigth^ cells, which are known for their immunoregulatory functions [18], could impair the NK-mediated modulation of adaptive immunity and contribute to a pro-inflammatory milieu. HVEM expression was higher on classical monocytes in patients who exhibited disability progression. As HVEM acts as an activator of monocytes [19,20], this suggests an early activation of the innate immune response in patients experiencing disability worsening. Taken together, this immune profile suggests a maladaptive immune response that may underlie disease progression in patients not initially recognized as having HAMS. Importantly, these results suggest that blood-based immunophenotyping can reveal underlying immune patterns linked to poor prognosis in MS. Incorporating these biomarkers into early risk assessment strategies may enhance the ability to accurately identify patients who would benefit from the prompt initiation of high-efficacy treatments.

In a subsequent step, we analyzed patients who were initially diagnosed as non HAMS but nonetheless experienced disability progression over the 3-year period. These individuals exhibited lower expression of PD-1 on memory Th cells compared to those who remained stable. The percentage of PD-1^+^ memory Th cells demonstrated strong predictive value for stability, with an AUC of 0.840. A threshold of 3.98% yielded a sensitivity of 83.2% and a specificity of 71.4% for predicting non-progression. This reduction in PD-1 expression may reflect increased T cell activity and excessive immune activation [21], contributing to neuroinflammation and subsequent disability progression in MS. These findings position PD-1 expression on memory Th cells as a valuable indicator of genuine disease stability, enabling the identification of patients with favorable long-term outcomes and candidates for low-efficacy treatments, even when early clinical evaluations may not accurately reflect their prognosis.

Moreover, in our study we identified immune-based biomarkers that predict PIRA, which of particular importance as it represents a form of silent progression that often goes unrecognized in early stages [22], especially in patients who may have HAMS but lack overt relapses. In our study, CD28 expression on terminal effector CD8^+^ T cells and HVEM expression on plasmablasts were identified as robust predictors of PIRA, with high sensitivity and specificity of 83.3%. Although CD28 expression is generally reduced in terminal effector cells compared with memory cells, it is not completely lost [23]. We observed a small but detectable expression of CD28 on terminal effector cells in patients presenting RAW compared with those with PIRA, potentially reflecting the maintenance of the inflammatory microenvironment in these patients and, consequently, the persistence of activated CD8^+^ T cells. Identifying immunological markers associated with PIRA could enable earlier intervention with high-efficacy therapies in patients who are otherwise underestimated by conventional relapse-based monitoring, ultimately aiming to preserve neurological function and improve long-term outcomes.

This study has several limitations that should be acknowledged. First, although the overall cohort size was determined a priori based on statistical power calculations, some subgroup analyses, particularly those derived from the ls-HAMS and ls-non HAMS subgroups, may have limited the ability to detect subtle but biologically relevant differences in IC expression. Overall, the present work should be considered a pilot study that provides a foundational framework for hypothesis generation and future large-scale investigations.

Another important limitation of this study is the absence of direct mechanistic validation of the immune checkpoint-related associations identified. While the biological relevance of the HVEM–BTLA axis and CD28 loss has been supported by functional studies in experimental models and other immune-mediated conditions, the present work does not directly address the mechanistic role of HVEM downregulation on effector Th cells or the functional consequences and potential reversibility of CD28 loss on naïve CD8^+^ T cells in different disease stages. Therefore, the observed associations should be interpreted as biologically plausible but correlative. Moreover, further validation in larger, independent cohorts, ideally across multiple geographically and demographically diverse centers, will be necessary to establish the diagnostic or prognostic utility of these biomarkers.

An additional limitation of this study is the limited ethnic diversity of the study population. Most of participants were of Caucasian background, resulting in a relatively homogeneous cohort. This lack of diversity may limit the generalizability of the findings to populations with different genetic backgrounds, environmental exposures, or immune profiles. Consequently, the applicability of the identified immune checkpoint-related signatures to more diverse patient populations remains to be determined and will require validation in cohorts with broader racial and ethnic representation.

## 4. Materials and Methods

### 4.1. Study Design

This is a clinical, observational, prospective, transversal and pilot study that includes patients with a diagnosis of RRMS, according to McDonald criteria [6], from June 2021 to December 2024. Patients with MS were stratified into two main groups based on disease duration:

1. Newly diagnosed and treatment-naïve (early-stage MS patients): These patients were newly diagnosed and had not yet received any DMT at the time of sampling. Patients were further subdivided according to disease activity:
  1.1. Early high-activity MS (e-HAMS): Males or females > 18 years recently diagnosed with MS who have not yet received DMT and exhibit the presence of at least two of the following poor prognostic factors: ≥20 lesions on MRI, ≥2 spinal/brainstem lesions, ≥2 gadolinium-enhancing lesions, ≥2 relapses per year, or EDSS ≥ 3.
  1.2. Early non-high-activity MS (e-non HAMS): Males or females > 18 years who are recently diagnosed, treatment-naïve MS patients who have experienced one or fewer of the poor prognostic factors defined in the previous group.
2. Long-standing MS patients (long-standing disease): Males or females over 18 years of age with MS who had received at least one prior DMT and had already completed the washout period required in the product information.
  2.1. Long-standing high-activity MS (ls-HAMS): Males or females > 18 years with long-standing MS who had previously failed treatment, as evidenced by relapses, activity on MRI, or disability progression, despite prior therapy.
  2.2. Long-standing non-high-activity MS (ls-non HAMS): Males or females > 18 years with long-standing MS who had previously received DMT and showed no signs of high disease activity at the time of inclusion. All patients had discontinued treatment for reasons other than therapeutic failure (risk of side effects, pregnancy, or patient choice).

None of the study participants may meet the following exclusion criteria: patients with progressive MS, current addiction to drugs or alcohol, or those suffering from a severe concomitant illness with an unfavorable short-term prognosis or another autoimmune disease. Pregnant or breastfeeding women and patients participating in pharmacological treatment trials were also excluded.

### 4.2. Ethical Statement

The study was approved by the Research Ethics Committee at La Paz University Hospitalon 27 January 2022 (PI-4675) and all patients provided their informed consent. All data processing was conducted within the framework of Spanish Law 14/2007, of 3 July, on Biomedical Research.

### 4.3. Demographic and Clinical Data

The following demographic data and clinical characteristics of the patients were collected during the study: sex, age, disease duration, lesions on MRI, lesions in the spinal cord or the brainstem on MRI, gadolinium-enhancing lesions, relapse ratio (relapses per year), EDSS score, and response to previous treatment (Table 1).

Disability progression over the three years was also analyzed, defined as an increase of 1.5 points in the EDSS score for patients with a baseline EDSS of 0; an increase of 1.0 point for those with baseline scores between 1.0 and 5.5; and an increase of 0.5 points for baseline EDSS scores of 6.0 or higher, measured at 36 months compared to baseline. Additionally, among patients who experienced disability progression, PIRA and RAW were assessed at 3 years. All clinical evaluations and outcome measurements were performed by experienced neurologists.

### 4.4. Blood Sample Processing

For newly diagnosed, treatment-naïve early-stage MS patients, blood samples were collected at the time of diagnosis, prior to the initiation of their first DMT. For long-standing MS patients, samples were collected after the discontinuation of their previous treatment, following an appropriate washout period based on the established clinical guidelines and the respective drug’s technical data sheet.

### 4.5. Neurofilament Light Chain Quantification

Plasma levels of NfL were measured using a chemiluminescence immunoassay on the Lumipulse G600 II platform (Fujirebio, Tokyo, Japan).

### 4.6. Immunophenotypic Profiling of Immune Cell Subpopulations

To identify the specific subpopulations that govern high activity, a characterization of the immune subset proportions was conducted and compared across different groups through spectral flow cytometry. For this purpose, peripheral blood mononuclear cells (PBMCs) were thawed, washed, and directly processed for staining. Cell viability was determined using a Live and Dead viability dye, and only samples with ≥80% viability were included in the analysis. A total of 21 subpopulations and their IC expressions were studied using the Cytek™ Aurora spectral flow cytometry (Cytek Biosciences, Fremont, CA, USA) and the resulting data were subsequently analyzed with FlowJo™ software v10.10 (BD, Ashland, OR, USA) as previously described [12].

### 4.7. Analysis of High Dimensional Data Using t-SNE

Ten thousand events from five random patients belonging to each study group were concatenated. The T-distributed stochastic neighbor embedding (t-SNE) algorithm was then applied using FlowJo^TM^ software v10.10 (BD, Ashland, OR, USA), focusing on the differentiation parameters of cell populations. In the resulting dimensional plot, the islands were analyzed to identify the cell subset. Once identified, IC marker expression analyses were performed for each population in each study group.

### 4.8. Statistical Analysis

The sample size for this pilot study was estimated using G*Power 3.1 (Heinrich-Heine-Universität Düsseldorf, Düsseldorf, Germany) to achieve a statistical power of 80% and a significance level (α) of 0.05. Data are presented as mean ± standard deviation (SD). All collected variables were classified as either categorical or numerical prior to analysis. Statistical analyses were performed using custom scripts in R version 4.5.0 (R Core Team, Vienna, Austria), RStudio version 2025.05.0 (Posit PBC, Boston, MA, USA), and SPSS version 27.0 for Windows (IBM Corp., Armonk, NY, USA). Normality of continuous variables was assessed using either the Shapiro–Wilk test or the Kolmogorov–Smirnov test with Lilliefors correction, depending on the sample size. Variables not meeting the criteria for normal distribution were analyzed as non-normally distributed. For comparisons between two groups, Student’s *t*-test or Mann–Whitney U test was applied based on the data distribution. Categorical variables were compared using the Chi-square test or Fisher’s exact test, as appropriate. Where applicable, *p* values were adjusted using the Benjamini–Hochberg procedure to control the false discovery rate. For comparisons across more than two groups, one-way ANOVA or the Kruskal–Wallis test was used, followed by post hoc analyses using Tukey’s HSD or Dunn’s test, respectively. Correlations between variables were evaluated using Pearson or Spearman correlation coefficients, depending on the normality of the data. Multiple linear regression analysis was performed to evaluate the independent effects of predictor variables on the outcome variable. The predictive performance and discriminative ability of the final model were assessed using ROC curve analysis, with the AUC reported as a measure of model accuracy. All graphical representations were generated using GraphPad Prism v8 (GraphPad Software, San Diego, CA, USA). Statistical significance was defined as follows: *, *p* < 0.05; **, *p* < 0.01; ***, *p* < 0.001.

## 5. Conclusions

Our findings in this pilot study reveal two distinct panels of immune biomarkers: one capable of identifying patients who have high activity from the time of diagnosis and may benefit from the early initiation of high-efficacy DMTs, and another applicable to individuals with long-standing, treatment-refractory MS. In addition, we defined a set of progression-associated markers detectable at the outset of follow-up, enabling the timely recognition of patients at a heightened risk of disability accumulation. These results underscore the clinical utility of integrating peripheral immune profiling into diagnostic workflows and treatment algorithms, offering a more precise and individualized assessment of disease trajectory.

## Figures and Tables

**Table 1:** Demographic data.

	HAMS (*n* = 61)	Non HAMS (*n* = 38)	*p* Value
**Demographics**			
Wome,. *n* (%)	34 (54.8%)	21 (56.8%)	
Age, years	42.22 (10.56)	44.41 (9.16)	0.251
**Ethnicity,** *n* (%)			0.390
Caucasic	54 (88.5%)	37 (97.4%)	
Hispanic	5 (8.2%)	1 (2.6%)	
African	2 (3.3%)	-	
Early stage, *n* = 41, *n* (%)	26 (63.4%)	15 (36.6%)	
Long standing, *n* = 59, *n* (%)	35 (60.3%)	23 (39.7%)	
**Clinical data**			
Baseline expanded disability status scale	2.36 (2.27)	1.36 (1.77)	**0.011**
Baseline symbol digit modality test	41 (14.32)	50.83 (12.07)	**0.001**
3 years progression	24 (39.9%)	10 (27.8%)	
3 years progression independent of relapse activity	18 (75.0%)	10 (100%)	
**Treatments received**			
Interferon, *n* (%)	2 (3.3%)	5 (13.2%)	
Teriflunomide, *n* (%)	4 (6.6%)	2 (5.3%)	
S1P receptor antagonists, *n* (%)	1 (1.6%)	-	
Dimethyl fumarate, *n* (%)	5 (8.2%)	9 (23.7%)	
Cladribine, *n* (%)	17 (27.9%)	14 (36.8%)	
Natalizumab, *n* (%)	20 (32.8%)	2 (5.3%)	
Anti-CD20, *n* (%)	12 (19.7%)	6 (15.8%)	
**Previous treatments received**			
Interferon, *n* (%)	8 (22.9%)	6 (26.1%)	
Teriflunomide, *n* (%)	3 (8.6%)	3 (13.0%)	
Alemtuzumab, *n* (%)	2 (5.7%)	-	
Dimethyl fumarate, *n* (%)	13 (37.1%)	4 (17.4%)	
Glatiramer acetate, *n* (%)	2 (5.7%)	6 (26.1%)	
Cladribine, *n* (%)	1 (2.9%)	-	
Natalizumab, *n* (%)	5 (14.3%)	3 (13.0%)	
Anti-CD20, *n* (%)	1 (2.9%)	1 (4.3%)	

All values are mean (SD) unless otherwise noted. Values reaching statistical significance are indicated in bold, dash indicate this variable is not found. We compared the distribution of patients with HAMS and non HAMS status across different previously received treatments. No statistically significant association was found between treatment group and HAMS/non HAMS status (Chi-square test, χ^2^ (7) = 8.43; *p* = 0.296). Mann–Whitney U test for continuous variables and Fisher’s exact test for categorical variables were employed to determine statistically significant differences between groups. Abbreviations: N, number; SD, standard deviation; HAMS, high-activity MS.

**Table 2:** No significant differences in immune profiles across prior treatment groups.

	Interferon	Teriflunomide	Dimethyl Fumarate	Glatiramer Acetate	*p* Value
B cells	73.62 ± 12.94	64.62 ± 17.20	70.56 ± 9.63	68.42 ± 19.00	0.605
HVEM effector Th	1.00 ± 0.68	0.88 ± 0.61	1.07 ± 1.02	0.49 ± 0.30	0.409
Memory CD8^+^ T	16.00 ± 8.47	12.23 ± 8.82	11.34 ± 4.94	12.35 ± 9.35	0.455
CD28 naïve Treg	99.2 ± 1.47	99.87 ± 0.15	99.49 ± 0.57	99.77 ± 0.57	0.253
CD28 naïve CD8^+^ T	2.19 ± 1.49	2.19 ± 1.84	1.50 ± 1.59	2.50 ± 3.16	0.390
HVEM classical monocytes	0.70 ± 0.61	0.58 ± 0.40	0.7 ± 0.41	0.72 ±0.34	0.782
CD28 terminal effector CD8^+^	3.90 ± 4.95	2.90 ± 2.78	3.87 ± 7.49	3.27 ± 5.02	0.509
CD70 NK^bright^	1.00 ± 0.89	0.84 ± 0.80	0.73 ± 0.54	0.41 ± 0.22	0.540
PD-1 memory Th	6.29 ± 2.26	4.82 ± 2.97	6.06 ± 3.54	5.71 ± 2.54	0.486
HVEM plasmablasts	6.13 ± 4.62	2.12 ± 0.73	3.27 ± 3.30	1.99 ± 2.44	0.127

All values are mean ± SD. ANOVA or Kruskal–Wallis tests were used to compare more than two groups. Post hoc comparisons were conducted using Tukey HSD or Dunn’s test to assess pairwise differences between groups.

**Table 3:** Multivariate linear regression analysis of predictors of PIRA.

Variable	Initial Model		Final Model	
	OR (95% CI)	*p* Value	OR (95% CI)	*p* Value
CD28 on terminal effector CD8^+^ T cells	1.548 (0.986, 2.428)	0.057	1.565 (1.081, 2.265)	**0.018**
CD80 on memory Treg	1.236 (0.168, 9.100)	0.836	-	-
CTLA-4 on memory B cells	0.570 (0.208, 1.562)	0.275	-	-
HVEM on plasmablast	1.349 (0.943, 1.929)	0.102	1.244 (0.977, 1.582)	0.076

Values reaching statistical significance are indicated in bold, dash indicate this variable is not foundFor each one-unit increase in CD28 expression on terminal effector CD8^+^ cells, the odds of experiencing relapse-associated progression increase by 56.5% (OR−1 = 1.565−1 = 0.565). For each one-unit increase in HVEM expression on plasmablasts, the odds of experiencing relapse-associated progression increase by 24.4% (OR-1 = 1.244-1 = 0.244).

## Data Availability

The data that support the findings of this study are available from the corresponding author upon reasonable request.

## Abbreviations

The following abbreviations are used in this manuscript:

CNS	central nervous system
DMTs	disease-modifying therapies
EDSS	expanded disability status scale
e-HAMS	early high-activity MS
e-non HAMS	early non-high-activity MS
HAMS	highly active multiple sclerosis
ICs	immune checkpoint
ls-HAMS	long-standing high-activity MS
ls-non HAMS	long-standing non-high-activity MS
MRI	magnetic resonance imaging
MS	multiple sclerosis
NfL	neurofilament light chain
PBMCs	peripheral blood mononuclear cells
PIRA	progression independent of relapse activity
PMS	progressive MS
PPMS	primary progressive MS
RAW	relapse associated worsening
ROC	receiver operating characteristic
RRMS	relapsing-remitting MS
SPMS	secondary progressive MS
t-SNE	T-distributed stochastic neighbor embedding
Th	T helper cell

## References

[1] Bierhansl L., Hartung H.-P., Aktas O., Ruck T., Roden M., Meuth S.G. (2022). Thinking outside the box: Non-canonical targets in multiple sclerosis. Nat. Rev. Drug Discov..

[2] Friese M.A., Schattling B., Fugger L. (2014). Mechanisms of neurodegeneration and axonal dysfunction in multiple sclerosis. Nat. Rev. Neurol..

[3] Kamińska J., Koper O.M., Piechal K., Kemona H. (2017). Multiple sclerosis etiology and diagnostic potential. Postepy Hig. Med. Dosw..

[4] Díaz C., Zarco L.A., Rivera D.M. (2019). Highly active multiple sclerosis: An update. Mult. Scler. Relat. Disord..

[5] Iacobaeus E., Arrambide G., Amato M.P., Derfuss T., Vukusic S., Hemmer B., Tintoré M., Brundin L. (2020). Aggressive multiple sclerosis (1): Towards a definition of the phenotype. Mult. Scler..

[6] Meca-Lallana J., Yélamos S.M., Eichau S., Llaneza M., Martínez J.M., Martínez J.P., Lallana V.M., Torres A.A., Torres E.M., Río J. (2024). Consensus statement of the Spanish Society of Neurology on the treatment of multiple sclerosis and holistic patient management in 2023. Neurologia.

[7] Meca-Lallana J., García-Merino J.A., Martínez-Yélamos S., Vidal-Jordana A., Costa L., Eichau S., Rovira À., Brieva L., Agüera E., Zarranz A.R.-A. (2021). Identification of patients with relapsing multiple sclerosis eligible for high-efficacy therapies. Neurodegener. Dis. Manag..

[8] Yang J., Hamade M., Wu Q., Wang Q., Axtell R., Giri S., Mao-Draayer Y. (2022). Current and future biomarkers in multiple sclerosis. Int. J. Mol. Sci..

[9] Mehlhop-Williams E.R., Bevan M.J. (2014). Memory CD8+ T cells exhibit increased antigen threshold requirements for recall proliferation. J. Exp. Med..

[10] Seals M.R., Moran M.M., Leavenworth J.D., Leavenworth J.W. (2022). Contribution of dysregulated B-cells and IgE antibody responses to multiple sclerosis. Front. Immunol..

[11] del Rio M.-L., Fernandez-Renedo C., Scheu S., Pfeffer K., Shintani Y., Kronenberg M., Chaloin O., Schneider P., Rodriguez-Barbosa J.-I. (2014). Therapeutic blockade of LIGHT interaction with herpesvirus entry mediator and lymphotoxin β receptor attenuates in vivo cytotoxic allogeneic responses. Transplantation.

[12] López-Molina M., Iglesias G.T., María-Diez G.S.d.S., Valentín-Quiroga J., Laso-García F., Gallego R., Pozo-Novoa J., Chamorro B., López-Collazo E., Puertas I. (2025). Immune checkpoint-based biomarkers for therapeutic response in patients with multiple sclerosis. Front. Immunol..

[13] Monreal E., Fernández-Velasco J.I., Álvarez-Lafuente R., de la Maza S.S., García-Sánchez M.I., Llufriu S., Casanova B., Comabella M., Martínez-Yélamos S., Galimberti D. (2024). Serum biomarkers at disease onset for personalized therapy in multiple sclerosis. Brain.

[14] Bănică L., Vlaicu O., Jipa R., Abagiu A., Nicolae I., Neaga E., Oțelea D., Paraschiv S. (2021). Exhaustion and senescence of CD4 and CD8 T cells that express co-stimulatory molecules CD27 and CD28 in subjects that acquired HIV by drug use or by sexual route. Germs.

[15] Shui J.W., Steinberg M.W., Kronenberg M. (2011). Regulation of inflammation, autoimmunity, and infection immunity by HVEM-BTLA signaling. J. Leukoc. Biol..

[16] Kim W., Bae E., Kang Y., Bae H., Hong S.H., Lee J.Y., Park J., Kwon B.S., Suk K., Lee W. (2006). Glucocorticoid-induced tumour necrosis factor receptor family related protein (GITR) mediates inflammatory activation of macrophages that can destabilize atherosclerotic plaques. Immunology.

[17] Smolders J., Hamann J. (2022). Programmed cell death protein 1-positive CD8+ T cells in multiple sclerosis: Exhausted fighters or peacekeepers. Neurol. Neuroimmunol. Neuroinflamm..

[18] Roe K. (2024). Immunoregulatory natural killer cells. Clin. Chim. Acta.

[19] Zhang Q., Vignali D.A.A. (2016). Co-stimulatory and co-inhibitory pathways in autoimmunity. Immunity.

[20] Kang Y.M., Kim S.Y., Kang J.H., Han S.W., Nam E.J., Kyung H.S., Park J.Y., Kim I.S. (2007). LIGHT up-regulated on B lymphocytes and monocytes in rheumatoid arthritis mediates cellular adhesion and metalloproteinase production by synoviocytes. Arthritis Rheum..

[21] Jubel J.M., Barbati Z.R., Burger C., Wirtz D.C., Schildberg F.A. (2020). The role of PD-1 in acute and chronic infection. Front. Immunol..

[22] Sharrad D., Chugh P., Slee M., Bacchi S. (2023). Defining progression independent of relapse activity (PIRA) in adult patients with relapsing multiple sclerosis: A systematic review. Mult. Scler. Relat. Disord..

[23] Topp M.S., Riddell S.R., Akatsuka Y., Jensen M.C., Blattman J.N., Greenberg P.D. (2003). Restoration of CD28 expression in CD28- CD8+ memory effector T cells reconstitutes antigen-induced IL-2 production. J. Exp. Med..

